# Supplementary material for: Visualization-enhanced under-oil open microfluidic system for in situ characterization of multi-phase chemical reactions
Source: Nat Commun. 2024 Feb 7;15:1155. doi: 10.1038/s41467-024-45076-7 (PMC10850056; doi:10.1038/s41467-024-45076-7)
Supplement: Supplementary file 1 — Supplementary Information [file 41467_2024_45076_MOESM1_ESM.pdf]

Supplementary Information for

Visualization-enhanced Under-oil Open Microfluidic System for *in situ* Characterizations of Multi-phase Chemical Reactions

Qiyuan Chen[1], Hang Zhai[2], David J. Beebe[3,4,5], Chao Li*[3], and Bu Wang*[1,2]

*To whom correspondence may be addressed.

email: bu.wang@wisc.edu (Bu Wang); cli479@wisc.edu (Chao Li)

This PDF file includes:

Supplementary Notes

- **Section 1- Mapping V-UOMS micro-channel using confocal Raman spectroscopy**
- **Section 2 - Passive Pumping Dynamic Flow Analysis**

Supplementary Figure S1 – S6

Supplementary References

Supplementary Notes

Section 1 - Mapping V-UOMS micro-channel using confocal Raman spectroscopy

We reconstructed the cross-section of a micro-channel in V-UOMS using the established boundary scanning with Raman spectroscopy (Fig. S1). A micro-channel with a channel width (*W*) of 0.5 mm that connects two 3 mm-diameter micro-spots was used in this demonstration, with a channel length (*L*) of 10 mm, written in the format of 3-3-*L*10-*W*0.5, in mm. Similar to the boundary scanning (Fig. 3), the micro-channel was filled with 10 μL 1M $NaNO_3$ aqueous solution and then scanned near the micro-channel mid-point in the Y-Z plane (channel cross-section; see Fig. S1a). The cross-section heat map is reconstructed based on the intensity of $v(NO_3^-)$ (1020-1080 cm^{-1} window; Fig. S1b) and silicone oil (1220-1280 cm^{-1} window; Fig. S1c) respectively. In this cross-section reconstruction, we utilized the 50% intensity of the maximum Raman signal intensity as the threshold to determine the estimated boundary between the oil and the media phases, to evaluate the shape of the micro-channel (i.e., width-to-height ratio). The channel cross-section can be identified with both phases (media and oil) and the results are consistent. The channel width-to-height ($W_{channel}/H_{channel}$) ratio from Raman reconstruction was found to be around 6:1. By applying this technique, it is possible to monitor the dynamic channel shape change in real-time, which is essential to quantify the fluid dynamics and mass transport in multi-phase chemical reactions.

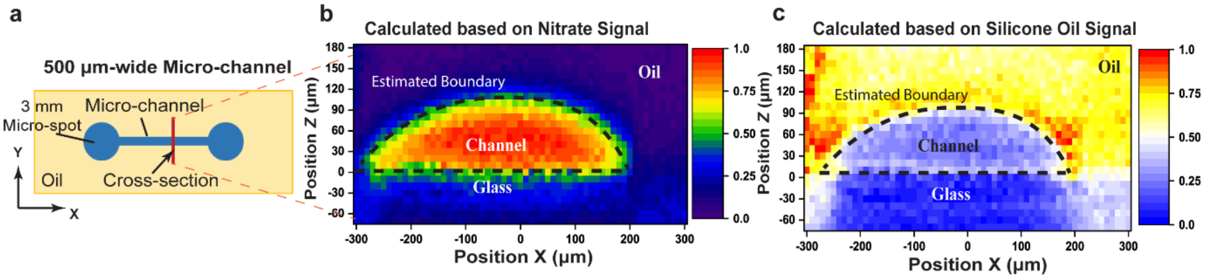

Fig. S1 Micro-channel cross-section reconstruction using Raman spectroscopy. a, Schematic diagram of the cross-section location and orientation relative to the microchannel. **b**, Heatmap of the cross-section calculated based on the $v(NO_3^-)$ peak. **c**, Heatmap of the cross-section calculated based on oil peak. The estimated boundary is defined by the 50% intensity of the maximum signal intensity.

Section 2 - Passive Pumping Dynamic Flow Analysis

As discussed in the main text, the gas evolution experiment in V-UOMS was performed with an under-oil micro-channel involving a lateral flow from the inlet spot to the outlet spot through the micro-channel driven by passive pumping - a convective flow driven by the pressure (e.g., Laplace pressure and/or hydrostatic pressure) difference between the inlet and outlet micro-drops connected by a micro-channel.[1] The micro-channel 3-3-*L*10-*W*0.5 - i.e., 10 mm in length, 500 µm in width, connecting two 3-mm diameter micro-spots as the inlet (for reagent loading) and outlet (for Raman signal collection) - was overlaid with silicone oil and then pre-filled with 10 µL 1 M Na_2CO_3 solution by under-oil sweep.

The Laplace pressure within the micro-drop, which is assumed to take a spherical cap shape, can be described by the Young-Laplace equation: [1,2]

$$\Delta P_{\mathrm{spot}} = 2\gamma_{\mathrm{oil/media}}/R_{\mathrm{spot}} \qquad (1)$$

where ΔP_{spot} is the Laplace pressure within the micro-drop, R_{spot} is the radius of curvature of the micro-drop, $\gamma_{\mathrm{oil/media}}$ is the interfacial tension of the oil-media interface (for silicone oil-water, $\gamma_{\mathrm{oil/media}}$ = 41.8 mN/m).[3]

For Micro-channel that is assumed to take a circular segment shape of the cross-section,

$$\Delta P_{\mathrm{channel}} = 2\gamma_{\mathrm{oil/media}}/R_{\mathrm{channel}} = \gamma_{\mathrm{oil/media}}/R_{\mathrm{channel\text{-}length}} + \gamma_{\mathrm{oil/media}}/R_{\mathrm{channel\text{-}width}} = \gamma_{\mathrm{oil/media}}/R_{\mathrm{channel\text{-}width}} \qquad (2)$$

where $\Delta P_{\mathrm{channel}}$ is the Laplace pressure within the micro-channel, R_{channel} is the radius of curvature of the micro-channel, $R_{\mathrm{channel\text{-}length}}$ is the radius of curvature of the micro-channel in the channel length direction, which is approximately infinitive, and $R_{\mathrm{channel\text{-}width}}$ is the radius of curvature of the micro-channel in the channel width direction ($R_{\mathrm{channel}} = 2R_{\mathrm{channel\text{-}width}}$).

The hydrostatic pressure is known as:

$$P_h = \rho g H \qquad (3)$$

where P_h is the hydrostatic pressure, ρ is the density of the media (approximately 1 g/mL) or 5-cSt silicone oil (0.91g/mL), g is the gravitational acceleration (9.8 N/kg), and H is the height of the liquid.

The V-UOMS device was placed under the confocal Raman microscope with the focal point set inside the outlet micro-drop (on the micro-spot) (Fig. 5a-b). To initiate the gas-evolution reaction, 4 μL 1 M H_2SO_4 was added to the inlet micro-drop through the oil overlay with a pipette. This added volume decreased the radius of curvature and therefore increased the Laplace pressure at the inlet micro-drop, triggering passive pumping from the inlet to the outlet. The entire process contains four stages: Stage (i) – Adding media by under-oil sweep, Stage (ii) - Reaching pressure equilibrium, Stage (iii) – Adding reagent to the inlet spot, and Stage (iv) – Passive pumping.

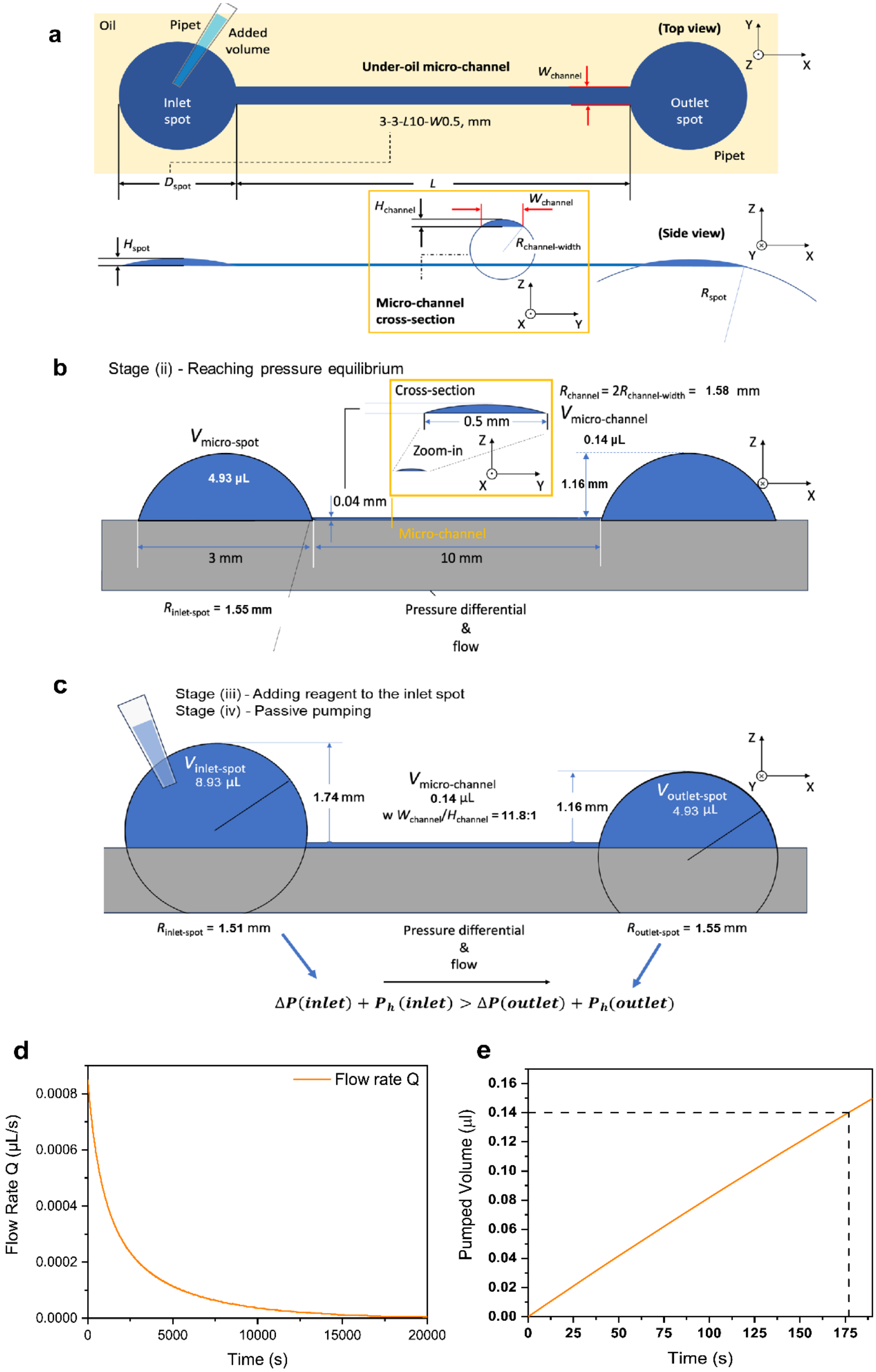
a
Oil
Pipet
Added volume
(Top view)
Under-oil micro-channel
$W_{channel}$
Inlet spot
Outlet spot
3-3-L10-W0.5, mm
Pipet
D_{spot}
L
$H_{channel}$
$W_{channel}$
H_{spot}
$R_{channel-width}$
(Side view)
Micro-channel cross-section
R_{spot}
b
Stage (ii) - Reaching pressure equilibrium
Cross-section
$R_{channel} = 2R_{channel-width} =$ 1.58 mm
0.5 mm
$V_{micro-channel}$
0.14 μL
Zoom-in
$V_{micro-spot}$
4.93 μL
1.16 mm
0.04 mm
Micro-channel
3 mm
10 mm
$R_{inlet-spot}$ = 1.55 mm
Pressure differential & flow
c
Stage (iii) - Adding reagent to the inlet spot
Stage (iv) - Passive pumping
$V_{inlet-spot}$ 8.93 μL
1.74 mm
$V_{micro-channel}$ 0.14 μL
w $W_{channel}/H_{channel}$ = 11.8:1
1.16 mm
$V_{outlet-spot}$ 4.93 μL
$R_{inlet-spot}$ = 1.51 mm
Pressure differential & flow
$R_{outlet-spot}$ = 1.55 mm
$\Delta P(inlet) + P_h(inlet) > \Delta P(outlet) + P_h(outlet)$
d
Flow rate Q
Flow Rate Q (μL/s)
Time (s)
e
Pumped Volume (μl)
Time (s)

Fig. S2 Passive pumping fluid dynamic analysis. a, An overview of the under-oil micro-spot and micro-channel (3-3-*L*10-*W*0.5). **b**, Key parameters and calculated geometry in Stage (i) and Stage (ii) i.e., -equilibrium after initial 10 μL of 1 M Na_2CO_3 solution was added. The channel width-to-height ratio at equilibrium is used in the calculations for Stage (iii) and (iv). **c**, Key parameters and calculated geometry for Stage (iii). Here, the gas-evolution experiment was initiated by adding an extra 4 μL of 1 M H_2SO_4 to the inlet micro-spot. **d,** Calculated results of the passive pumping process in Stage (iv). The flow rate Q decreased as the reagent was pumped from the inlet micro-drop to the outlet (which decreased the pressure difference). The whole process would take over 20,000 seconds to reach equilibrium. **e,** Calculated cumulative passive pumping volume for the initial 200 seconds.

Stage (i) - Adding media by under-oil sweep & Stage (ii) - Reaching pressure equilibrium

To perform under-oil sweep, a hanging drop of the 1 M $NaNO_3$ aqueous solution at the end of the pipet tip was dragged across the patterned surface with Double-ELR - i.e., under-oil water ELR (for the untreated surface with PDMS-silane monolayer) + under-water oil ELR (for the O_2 plasma-treated areas, e.g., the micro-spots and micro-channels).[1] 10 μL of the 1 M $NaNO_3$ solution was added to micro-spots and micro-channel. Driven by the pressure differential between the micro-channel and the micro-spots (Fig. S2b), the system reached pressure equilibrium until the pressures were balanced:

$$\Delta P_{spot} + P_{h\ (spot)} = \Delta P_{channel} + P_{h\ (channel)} \qquad (4)$$

where ΔP is the Laplace pressure, P_h is the hydrostatic pressure. Based on calculation, in order to maintain the pressure equilibrium, the channel had a $W_{\text{channel}}/H_{\text{channel}}$ ratio of 11.8:1.

Stage (iii) - Adding reagent to the inlet spot & Stage (iv) - Passive pumping

Then, 4 μL of 1 M H_2SO_4 was added to the inlet micro-drop (Supplementary Fig. 2c). After loading the reagent, the volume of the inlet micro-drop increased to 8.93 μL. The pressure difference between the inlet and the outlet micro-drops can be calculated as the sum of the Laplace pressure difference between the inlet and the oulet, 55.66 – 53.93 = 1.73 Pa.

The volumetric flow rate (Q) from inlet to outlet through the micro-channel is described by Poiseuille's law as:

$$Q = (P^*_{\text{inlet}} - P^*_{\text{outlet}})/R_h \qquad (5)$$

where P^* is the pressure within the micro-drop, R_h is the hydrodynamic resistance of the under-oil micro-channel. Here, R_h is calculated based on the following assumptions: (I) the fluid is incompressible, Newtonian, and in laminar flow with a non-slippery boundary;[3] and (II) the microchannels take only one non-slippery boundary (with substrate) – i.e., single plate shape model.[4] From the two plates shape model,[4] we assume flow through a single plate model only experiences half of the hydrodynamic resistance, leading to:

$$R_h = 6\mu L/(WH^3) \qquad (6)$$

where μ is the viscosity of the liquid. For 1 M Na_2CO_3 solution at room temperature,[5] $\mu = 13 \times 10^{-10}$ $N{\cdot}s/mm^2$. L is channel length, W is channel width, and H is channel height.

By implementing Equations (1) and (3) to (6), we could calculate the flow rate Q. The initial flow rate Q_0 was found to be 0.00085 μL/s.

As the liquid is pumped to achieve a new pressure equilibrium, the pressure difference between the inlet and outlet will decrease, so will the flow rate. The time evolution of the flow rate can be calculated using a finite difference approach, i.e., solving for Q_0 at small time intervals with the inlet/outlet pressure difference updated at each interval based on micro-drop volumes. Here, we used a time interval of 10 seconds. Specifically, at the ith time interval, the volume change ΔV from the last interval is given by $\Delta V = Q_i \times \Delta t$, where Q_i is the flow rate at the ith interval and Δt is the time internal, i.e., 10 seconds. After pumping for 10 seconds, the volume at the inlet becomes $V_{(inlet)new} = V_{(inlet)} – \Delta V$, and at the outlet $V_{(outlet)new} = V_{(outlet)} + \Delta V$. The new volumes produce a new flow rate, Q_{i+1}, which will then be used for the next time interval.

Results of this calculation are shown in Fig. S2d, e. The cumulative volume of the media that needs to

be pumped to transport SO_4^{2-} from the inlet to the outlet equals the volume of the micro-channel $V_{\text{micro-channel}}$ = 0.14 µL. Based on the calculation, pumping this amount of media would take 177 s. For comparison, it would take over 20,000 seconds to pump half of the added liquid volume to outlet micro-drop.

Supplementary Figures

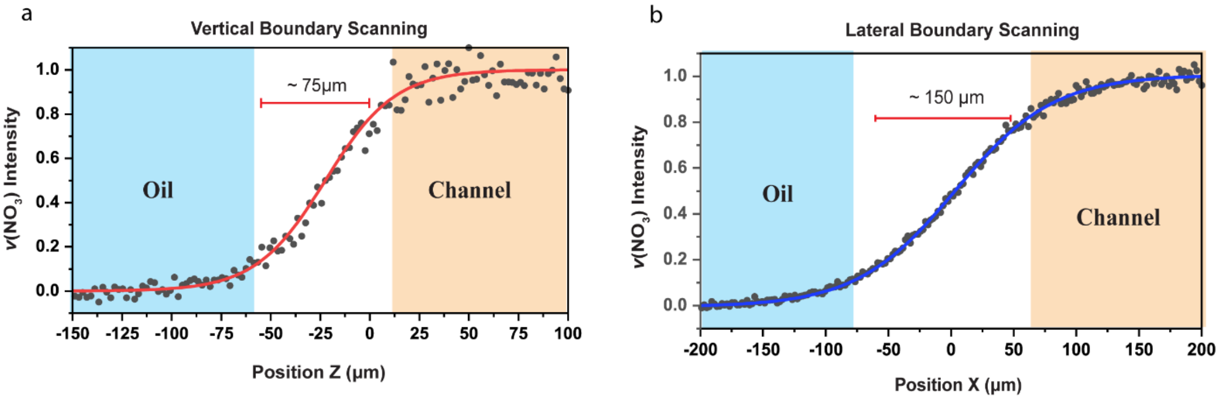

Fig. S3 Interfacial scanning in UOMS on Glass Slide. a, Vertical interfacial scanning; **b,** lateral interfacial scanning. The scattering plots are normalized NO_3^- intensity data, we then overplot the fitted sigmoid function respectively.

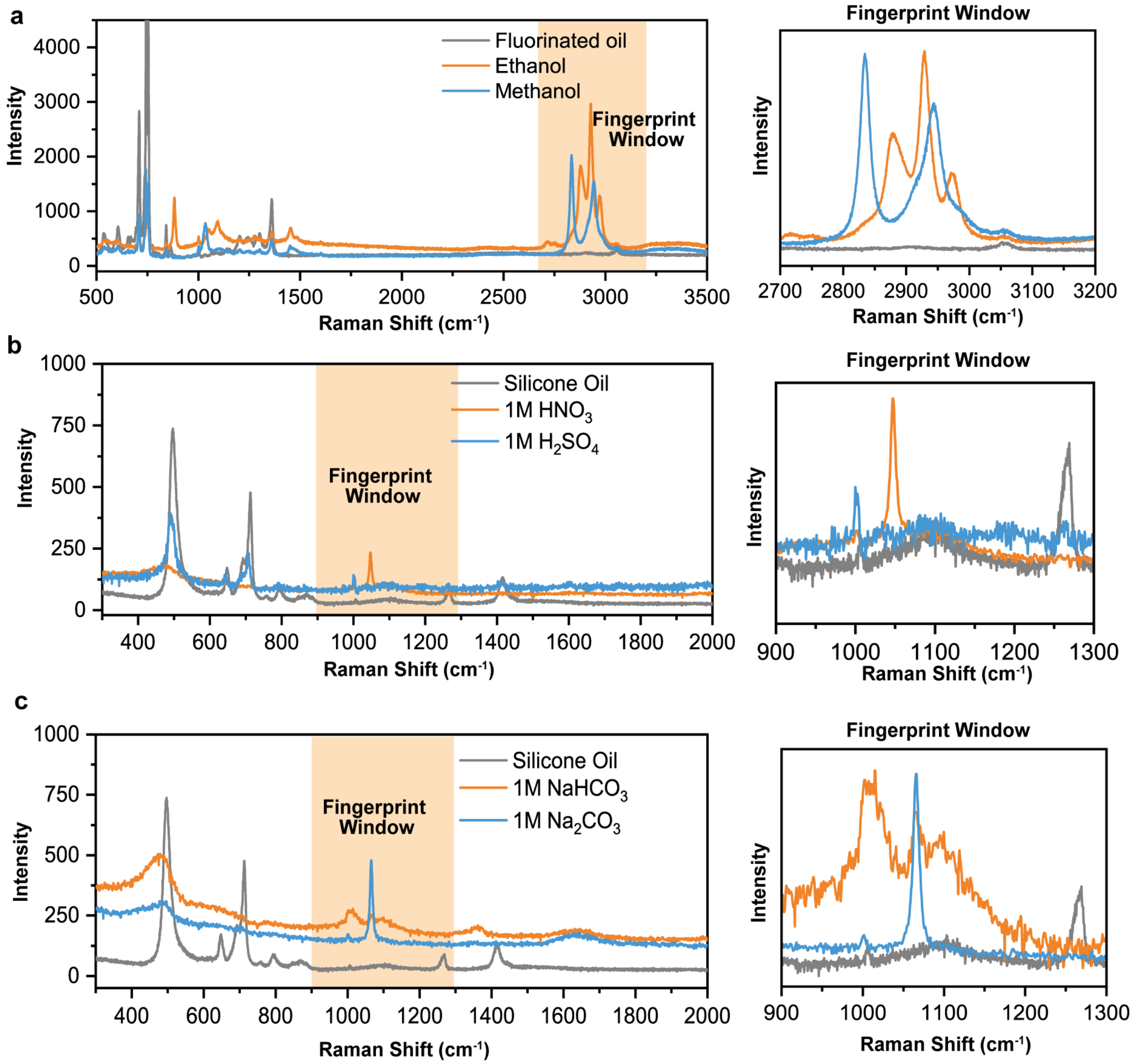

Fig. S4 Raman spectrums of sample media in three different environments. **a,** Organic solvents (here ethanol and methanol). **b,** pH < 1 (here 1M HNO_3 and 1M H_2SO_4). **c,** pH > 12 (here 1M $NaHCO_3$ and 1M Na_2CO_3)

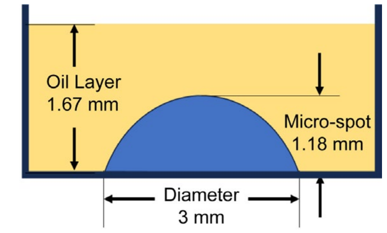

Fig. S5 Illustration of under-oil setups for CO_2 capture experiment. The micro-spot is filled with 5 μL 1M Na_2CO_3 and covered with 4 mL four types of oils [silicone oil (5 cSt), fluorinert FC-40, mineral oil, and silicone oil (100 cSt)]

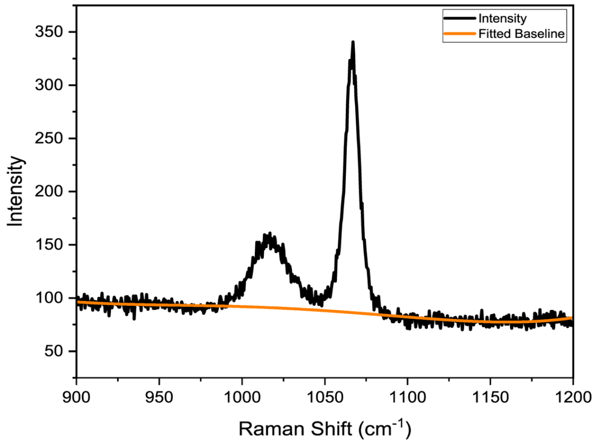

Fig. S6 Baseline fitting example. Background correction is carried out by a 4-th order polynomial fitting between the 900-1200 cm^{-1} window.

References

1. Li, C. *et al.* Under oil open-channel microfluidics empowered by exclusive liquid repellency. *Sci. Adv.* **6**, eaay9919 (2020).
2. Li, C. *et al.* Double-exclusive liquid repellency (double-ELR): an enabling technology for rare phenotype analysis. *Lab. Chip* **18**, 2710–2719 (2018).
3. Deroy, C. *et al.* Predicting flows through microfluidic circuits with fluid walls. *Microsyst. Nanoeng.* **7**, 93 (2021).
4. Bruus, H. *Theoretical microfluidics*. (Oxford University Press, 2008).
5. Correia, R. J., Kestin, J. & Khalifa, H. E. Viscosity and density of aqueous sodium carbonate and potassium carbonate solutions in the temperature range 20-90.degree.C and the pressure range 0-30 MPa. *J. Chem. Eng. Data* **25**, 201–206 (1980).
